# Vocational education phenomena research method

**DOI:** 10.1016/j.mex.2021.101537

**Published:** 2021-10-03

**Authors:** Linda Fauziyah Ariyani, Sri Umi Mintarti Widjaja, Hari Wahyono, Agung Haryono, Jack Febrian Rusdi, Chandra Bagus Agung Pratama

**Affiliations:** a Postgraduate Program, Faculty of Economics, Universitas Negeri Malang, Malang, Indonesia; b Faculty of Economics, Universitas Mulia, Balikpapan, Indonesia; c Jurusan Teknik Informatika, Sekolah Tinggi Teknologi Bandung, Bandung, Indonesia

**Keywords:** Vocational high school, Questionnaire design, Method stage, Indonesia

## Abstract

The vocational education implementation is one of the success factors for government to improve the quality of human resources, mainly toward producing skilled workers. Various researchers have studied the phenomena of vocational education. Unfortunately, studies on research methods in relation to vocational education phenomena are limited. Hence, this paper examined the research methods on the phenomena of vocational education, based on few case studies in Indonesia. Indonesia has many challenges as such Indonesia is a country with the fourth highest population globally, high level of unemployment, relatively rapid growth of new autonomy, and most number of islands in the world. In fact, Indonesia has different characteristics and needs of human resources from each region.

• This article discusses the methods to explore the phenomena of vocational education through case studies in Indonesia.
• This method article discusses the division of question groups and deployment of questionnaires through the referral and stages of research.
• This method article is the alternative basis for preparing research steps related to study the vocational education phenomena.

## Graphical abstract

**Figure:**
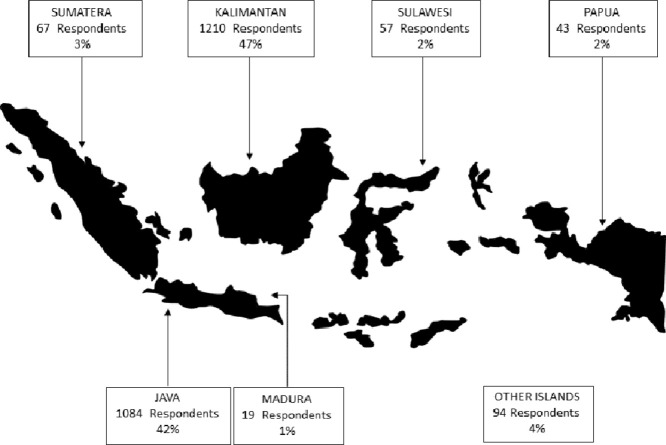

**Spesifications table**

**Table:** 

Subject Area	Economics/Business
More specific subject Area	Vocational Education
Method name	Vocational Education Phenomena Research Method
Name and reference of original method	P. Handayati, et al., “Does entrepreneurship education promote vocational students’ entrepreneurial mindset?,” Heliyon, vol. 6, no. 11, p. e05426, Nov. (2020), doi: 10.1016/j.heliyon.2020.e05426. [1]
Resource availability	J. F. Rusdi, “Vocational Education in Indonesia: Research Dataset - Mendeley Data,” Mendeley Data, vol. 1, (2021), doi: 10.17632/kvf9b3hs9f.1 [2]

## Introduction

Vocational education is a learning process organized by government to produce skilled workers [1]. A phenomenon study is a way to determine the field situation based on experience. Research on this phenomenon is important, especially to track the development of Vocational Education thus far, [3]. Various studies related to vocational education have been carried out in various fields, including History [4], evolution [5], developments [6], technology [7,8], regulation [9], strategy [10], employment [11], and outputs [12,13]. Indeed, studies on the phenomena of Vocational Education are essential in every country that organizes vocational education programs, such as East Germany [14], Tanzania [15], Thailand [16], Finland, Denmark and Sweden [17,18].

As for Indonesia, the implementation of Vocational Education begins from the secondary education known as Vocational High School (SMK). Vocational Education in Indonesia is unique, especially in terms of the size of the country's area, spreading in the form of an archipelago. The five major islands are Sumatra, Java, Kalimantan, Sulawesi, and Irian, with smaller islands totaling 17508 islands. In addition, Indonesia is a country with the fourth-largest population in the world. Vocational education is also an alternative solution for human resource problems and high unemployment rate in this country [19,20]. The different needs of each area trigger vocational education implementation in various disciplines [21], [22], [23].

The research question in this method article is “What is the method for researching the phenomena of vocational education?”, focusing on data collected from students and graduates of vocational education in secondary schools. Therefore, it is necessary to study methods related to the phenomena of vocational education.

Based on the studies on research methods related to the phenomena of Vocational Education, we were unable to find any article either through Science Direct or Google Scholar [2]. We, then, emphasized on case studies of Indonesia.

To ensure precise and accurate research data, we review the method articles. Among other things, the articles were intended to enrich the literature on the phenomena of vocational education, especially in terms of data collection through questionnaires and literature [2]. This study discussed how the research method was carried out in gaining richer insights of the Vocational Education phenomena, especially in Indonesia. The case studies were adopted based on the current and former students’ opinions in the questionnaires.

## Method details

**Fig. 1:**
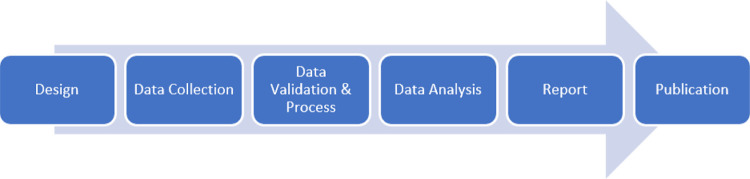
Research stages of the vocational education phenomena.

The method used in this study was carried out in several stages. The main stages are depicted in Fig. 1. The stages comprised Design, data collection, followed by data validation and process. The last three stages were Data Analysis, Reporting, and Publication.

### Research design

The first step was to design the research. Several activities were carried out at this stage such as determining research topics, reviewing literature, designing questionnaires, and designing strategies for distributing questionnaires.

#### Research topic

In this study, the topic chosen was on the phenomena of vocational education in Indonesia. This study revealed the views of current and former students experiencing the vocational education in Indonesia. The phenomena studied in this study were mainly to reveal the experience of Vocational Education from the students' point of view, including the reasons for choosing Vocational Education, students' views on the facilities and profile of the school, and the jobs of the graduates.

#### Literature study

Strong research must be supported by reliable research sources. Likewise, in Vocational Education research, the problems or gaps that have been raised by other researchers were examined. The areas of study and the ways Vocational Education research develops from time to time, including how the phenomena of Vocational Education are analyzed and conclusions generated from various parties were analyzed. Hence, this study was based on past studies examined by reviewing literature.

Furthermore, the activities being carried out were answering research questions through the results of other studies. The step taken was to review literature with the aim of exploring the current problems discovered by other researchers. Literature is the initial basis for research to see similar studies that have been carried out by other researchers. The references were the results of studies by other researchers that have been reviewed and published internationally, both through journals and proceedings. Literature sources were generated based on a search for research publications with ScienceDirect as the main source. Through this literature review, various developments related to research questions were produced, in addition to ascertaining whether the research topic raised had been previously studied. Thus far, research on the phenomena of vocational education in Indonesia has not been carried out.

#### Designing questionnaire

**Fig. 2:**
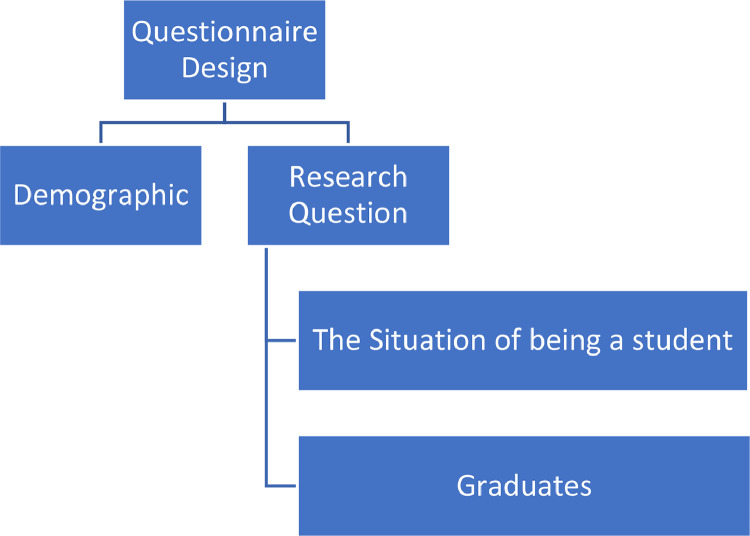
Questionnaire design of vocational education phenomena research.

The next step in designing this study was by designing a questionnaire [24]. Questionnaires are designed based on research needs that are tailored to the research topic and research questions. The questions asked in the questionnaire are divided into two main groups, namely the demographic information of the samples and the research questions themselves as shown in Fig. 2.

The demographic section of the questionnaire was the data of the participants [25], including the respondent's status, gender, regional origin, and age of the respondent. The position referred to whether the respondent was a student or graduate. Considering the vast possibility of distributing the questionnaire within Indonesia which is an archipelagic country, it is necessary to have the respondent's region of origin. The origin of this area was based on the island where the respondents resided. The questionnaire is shown in the repository [2].

Research questions are the contents of a questionnaire related to the situation faced by a student [26] or graduate [27]. Graduates only complete the questionnaire based on the case when they graduated from vocational high school.

#### Questionnaire distribution strategy

**Fig. 3:**
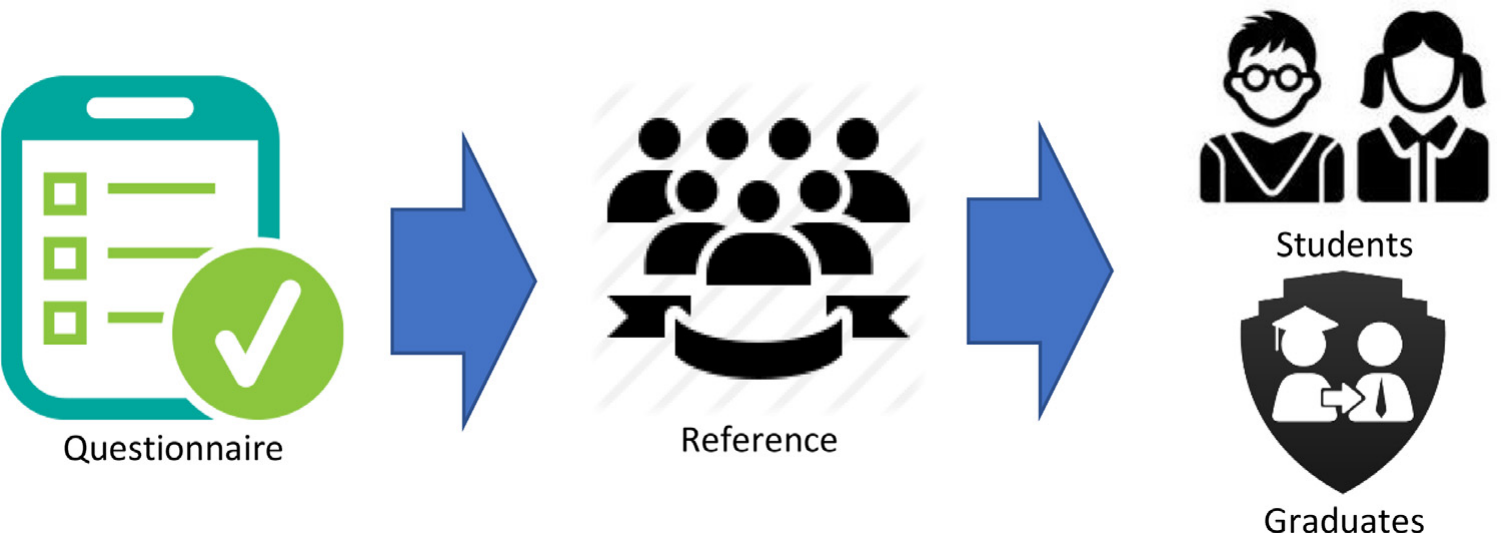
Questionnaire distribution strategy on vocational education phenomena research.

Given the wide coverage area and level of vocational education in Indonesia, and for this questionnaire to reach students and graduates of vocational education, a questionnaire dissemination strategy was needed. Questionnaire distribution was done through WhatsApp and Telegram media. In this method study, we invited participants through community groups closely related to Vocational Education in Indonesia. It is through this community group that this study reached the participants. Fig. 3 describes the strategy used in distributing the questionnaires.

Issues in the questionnaire distribution process included the access to students and graduates who scattered in various islands in Indonesia. Hence, the strategy of distributing questionnaires was carried out through different potential groups. The possible groups for distributing this questionnaire, with the main targets to complete the questionnaire, are described in Table 1.

**Table 1:** Distribution of invitations to fill out the questionnaire on vocational education phenomena research.

No	Group	Main Target	Island/Region
1	Association of high school principals	Student & Graduates	Kalimantan, Java, and Papua
2	Catholic School Association	Student & Graduates	Indonesia
3	Professional organization	Student & Graduates	Java and Kalimantan
4	National lecturer group	Student & Graduates	Indonesia
5	Student parent group	Student	Java and Kalimantan
6	SMK graduate's association	Graduates	Java and Kalimantan
7	Student Council Leader Group	Student	Kalimantan

#### Data collection

**Fig. 4:**
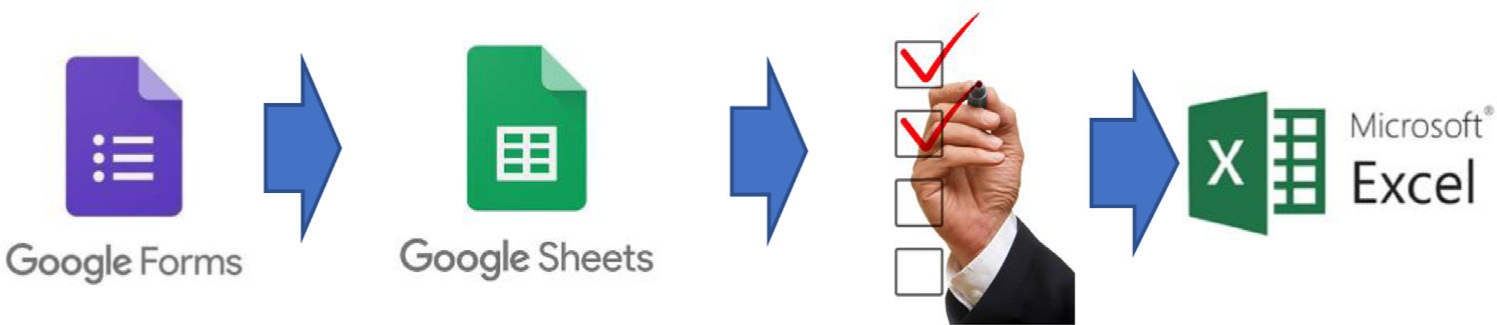
Data collection stage of the vocational education phenomena research.

The data collection was carried out from January 9, 2019, to August 5, 2019. Google Form was used for the data collection. Google Form stored data in the Google Sheets. 2578 forms were returned. The researcher reviewed the data, converted and saved them in a Microsoft Excel format file. The data stored in Microsoft Excel were used for further data processing in the next stage. Fig. 4 illustrates the processes carried out in the Data Collection Stage.

### Data validation and processing

**Fig. 5:**
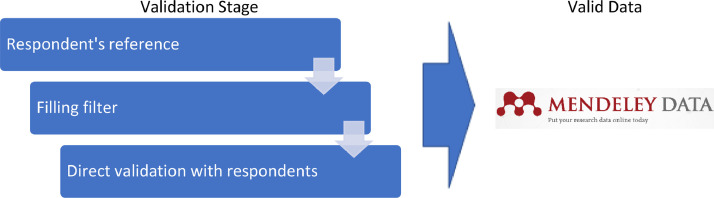
Data validation process to get valid data in vocational education phenomena research.

In any research, it is critical to consider the level of data validation toward quality data processing. The process of obtaining data with high validity was also considered in this study. To increase the level of validity of the data collected, each data entered was validated. Validation was carried out in three stages, namely based on respondent references, filling filters, and direct validation with respondents. Validation based on respondent references indicated that only respondents through a predetermined community group get the link to fill out the questionnaire. Meanwhile, the form stated that only students and graduates were allowed to fill out this questionnaire in terms of the filling filter. At the same time, direct validation with respondents signified the process of direct contact with respondents to ensure the data accuracy. Direct validation with respondents was carried out for certain data were deemed necessary to be verified with respondents directly. Respondents were contacted via WhatsApp communication media according to the contact number entered by the respondents concerned. Fig. 5 describes the data validation process toward obtaining valid data.

From the results of the data validation, four data were invalid. The inconsistency of the data entered caused the data to be invalid. For example, the respondent stated that his status as a student, but on the other hand, he mentioned information that identified him as an alumnus through his work experience, and the respondent could not be contacted by the researcher. The final data processed for analysis were 2574.

**Fig. 6.**
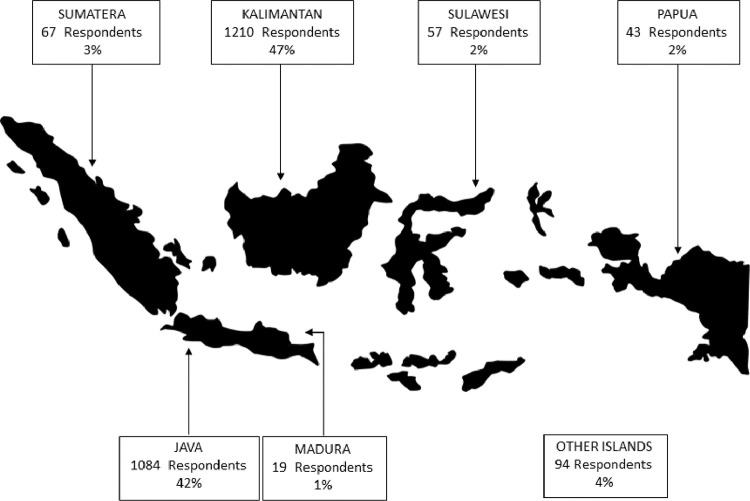
Respondence distribution.

The data collected are shown in Table 2. The number of secondary students were 1348 whereas 1226 were school graduates. In terms of gender, 1152 were male and 1422 female respondents. In terms of regional origin, most respondents were from the three largest islands namely Kalimantan (1210), Java (1084), and Sumatra (67), as shown in Fig. 6. In addition, most respondents were below 21 years old, comprising 1770 respondents. Table 2 demonstrates the respondence's demographic information.

**Table 2:** Questionnaire respondent information of vocational education phenomena research.

No	Name	Description	Respondent	%
1	Status	SMK Students	1348	52%
		SMK Graduates	1226	48%
2	Gender	Female	1152	45%
		Male	1422	55%
3	Origin	Kalimantan islands	1210	47%
		Java islands	1084	42%
		Sumatera islands	67	3%
		Papua islands	43	2%
		Sulawesi islands	57	2%
		Madura islands	19	1%
		Other islands	94	4%
4	Age (Y.O)	< 21	1770	69%
		21 - 25	294	11%
		26 - 30	200	8%
		31 - 35	196	8%
		36 - 40	56	2%
		41 - 45	28	1%
		> 45	30	1%

Data from the validation process were stored in the Mendeley repository. Respondents’ private data especially data related to the respondent's name and contact number [2] were not revealed.

### Data analysis

Data that passed the validation process were examined to obtain results. The results are the outputs of the data analysis, especially those related to research questions. The data analysis included reasons for choosing vocational education, school facilities and profiles, and the condition of school graduates after completing vocational education

#### Background for pursuing vocational education

**Fig. 7:**
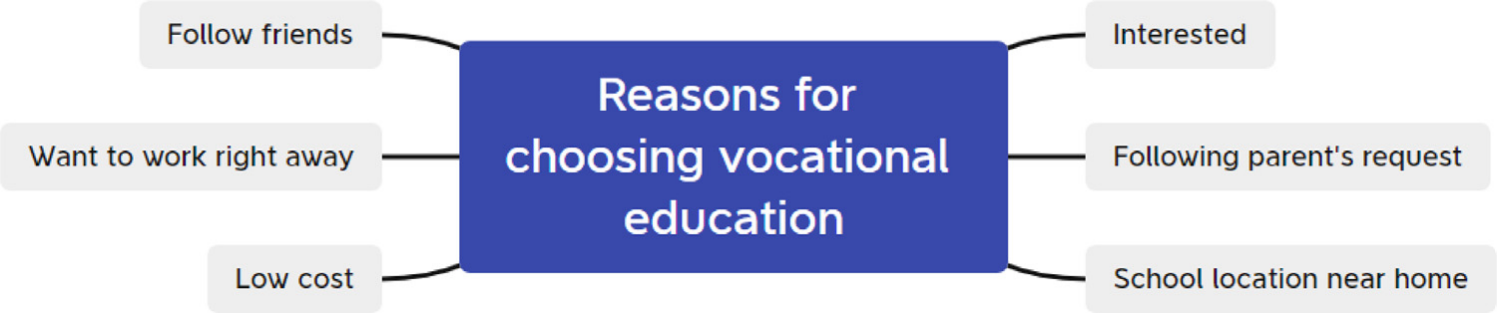
Reasons for choosing vocational education.

The questions comprised the background for pursuing Vocational education in terms of whether they matched their interests, followed the requests of their parents, the location of the school was close to where they lived, the costs were cheap, they wanted to work immediately, or because they wanted to follow their friends. The answers for this question are as shown in Fig. 7.

#### School assessment

**Fig. 8:**
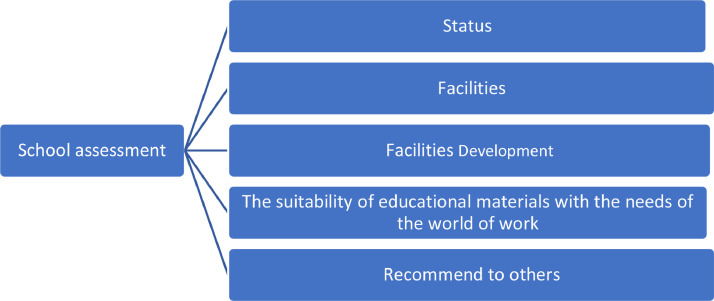
School assessment in vocational education.

Understanding school conditions based on the views of students and graduates needs to be analyzed, especially to carry out the control process of conditions in the field. What needs to be studied? Lots of information could be generated and needed to be monitored from the purview of the students and graduates regarding conditions in the field. Information was also extracted on the existing facilities and school profiles from the perspective of the students and graduates. Views of the school status, available facilities, facility development, material suitability with the work environment, and student and graduate willingness to recommend their school to other parties were also included. Fig. 8 shows the components of school assessment for Vocational Education.

#### Graduate assessment

**Fig. 9:**
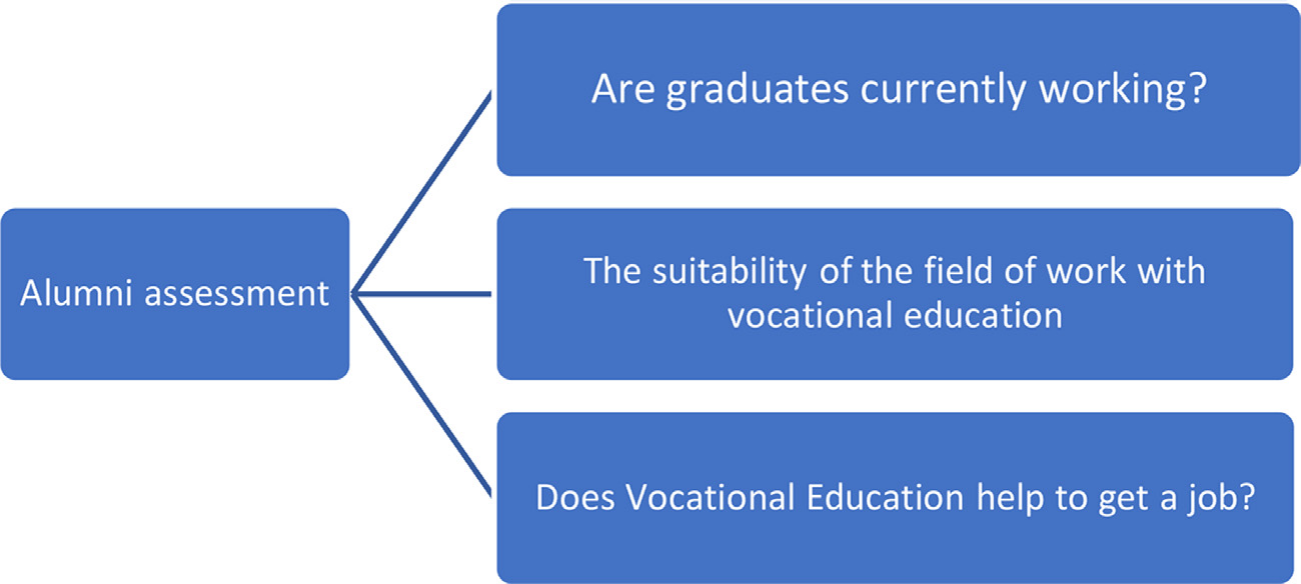
Education assessment for graduates.

This study also captured the views of graduates regarding their activities after graduation. In fact, this section examined several elements, including whether the graduates, upon answering the questionnaire, were already employed, the graduates’ field of work was in line with the vocational education learned and whether vocational education helped graduates in securing their jobs. In addition, the phenomenon of graduates’ ability to build and develop their own business was also pertinent. In a nutshell, the graduate perspectives should be heeded toward the Vocational Education development. Fig. 9 illustrates the components for alumni assessment.

### Research report

The research report is a scientific step carried out by researchers. This research report was in line with the applicable standards related to the publication of research results. This vocational education Phenomena Research was reported by covering the method and phenomena themselves. For this reason, this research is designed based on a scientific research report that follows the applicable system. The rules of writing, layout, and systematic procedures of the intended journals were to be adhered in order to pass the publication stage by the publisher.

### Research publications

The selected publications were sent to publishers of certain journals to be published. As a result of scientific research, the selected publication media are journals that have passed the peer review process, indicating that articles were reviewed by competent parties so that research articles were deemed appropriate and adhering to applicable rules. In terms of the discussion related to the method, we chose the MethodsX journal as the right medium to publish the research method article.

## Conclusion

The study of the method of Vocational Education in Indonesia is carried out in several structured stages by applying research design, data collection, validation, analysis, research report preparation, and publication of research results. The steps presented in this study can serve as an illustration for other research, especially to examine phenomena related to vocational education. Other researchers can use the research method applied in this study to develop a strategic research stage in implementing research methods in vocational education, specifically through a study using a questionnaire.

## Declaration of Competing Interest

None.
